# Transcriptome Analysis of DAMP-Induced Root Growth Regulation and Defense in Foxtail Millet

**DOI:** 10.3390/ijms26115175

**Published:** 2025-05-28

**Authors:** Hao Ye, Xinyu Xie, Qiongfang Fu, Sheng Zheng, Xunyan Liu, Shan Zhu

**Affiliations:** 1 College of Life and Environmental Sciences, Hangzhou Normal University, Hangzhou 311121, China; 2022210301105@stu.hznu.edu.cn (H.Y.); 2022210301123@stu.hznu.cn (X.X.); qiongfangf@163.com (Q.F.); liuxunyan@hznu.edu.cn (X.L.); 2 College of Life Sciences, Northwest Normal University, Lanzhou 730070, China; zhengsheng@nwnu.edu.cn

**Keywords:** foxtail millet, DAMP, pep1, transcriptomic, root development, gene expression, stress response

## Abstract

Foxtail millet (*Setaria italica* L.), a representative C4 species, is recognized for its efficient nutrient utilization and robust abiotic stress responses. However, the molecular mechanisms mediating its tolerance to biotic stresses are poorly understood. In this study, we investigated the root transcriptomic response of foxtail millet to the damage-associated molecular pattern (DAMP), the plant elicitor peptide 1 (Pep1). Transcriptome analysis of Pep1-treated roots identified 401 differentially expressed genes (DEGs), comprising 144 up-regulated and 257 down-regulated genes. Gene Ontology (GO) enrichment analysis revealed a significant enrichment of ‘peroxidase activity’. This finding was corroborated by DAB staining, which confirmed H_2_O_2_ accumulation, along with elevated malondialdehyde (MDA) levels, collectively indicating oxidative stress. Notably, Pep1 treatment also resulted in a marked up-regulation of the pathogenesis-related protein 1 (*PR1*) gene in leaves, suggesting the activation of systemic acquired resistance. Together, these results demonstrate that Pep1 triggers substantial transcriptional reprogramming in roots, induces oxidative stress, and activates systemic defense signaling in foxtail millet.

## 1. Introduction

Unlike motile animals, sessile plants are inherently constrained in their ability to escape environmental stressors, necessitating the evolution of complex regulatory networks for the precise perception and transduction of both biotic and abiotic stress signals. Small peptides function as critical extracellular signaling molecules in plants, facilitating intercellular communication and exerting regulatory control akin to classic phytohormones in modulating growth, development, and defense responses [1,2,3]. In *Arabidopsis thaliana*, pattern-triggered immunity (PTI) is activated upon recognition of conserved host and microbe-derived small peptides, such as CLAVATA3/ESR-RELATED (CLE), root meristem growth factor (RGF), plant elicitor peptide (Pep), flagellin epitope flg22, and elongation factor Tu epitope elf18, by cell-surface receptor-like kinases (RLKs) [4]. Beyond pathogen-associated molecular patterns (PAMPs), plant immunity is also activated by endogenous damage-associated molecular patterns (DAMPs), which are recognized by specific pattern recognition receptors (PRRs) to initiate PTI. Plant elicitor peptides (Peps), a class of DAMPs derived from PROPEP precursor proteins, play a significant role in plant immunity, acting as endogenous danger signals. In *Arabidopsis thaliana*, Peps are recognized by the leucine-rich repeat receptor kinases PEPR1 and PEPR2, triggering downstream immune signaling cascades [5,6]. Pep1, a well-characterized DAMP, regulates diverse biological processes, including immune responses and root development. Exogenous application of Pep1 has been demonstrated to enhance plant resistance against bacterial, fungal, and herbivore challenges [7,8,9,10]. The Pep-PEPR system is increasingly recognized as an important amplifier of basal plant innate immunity [11].

Roots exhibit a dual functionality, providing structural anchorage for aerial plant tissues and simultaneously mediating the acquisition of water and essential nutrients crucial for plant growth and development [12,13,14]. Root system architecture, encompassing length and structural organization, critically determines a plant’s adaptive capacity for nutrient acquisition and stress tolerance, especially drought, thereby influencing crop yield [15]. Root growth and development are regulated by both endogenous genetic factors and exogenous environmental signals [13,14]. In *Arabidopsis thaliana*, Pep-PEPR signaling has been shown to intersect with auxin-mediated pathways, influencing root cell expansion and differentiation during immune response activation. Additionally, Pep1 is implicated as a putative plant peptide hormone regulating root development in *Arabidopsis thaliana* and *Oryza sativa* [16,17]. These findings suggest a conserved role for Pep signaling in modulating root development, potentially linking stress perception with developmental plasticity.

Foxtail millet, as a C4 model system, exhibits significant adaptation to semi-arid and arid environments [18,19]. The inherent tolerance of foxtail millet to a range of severe abiotic stressors, combined with its relatively simple genome, establishes it as a valuable model system for studying abiotic stress resistance mechanisms in gramineous crops. Numerous studies have explored abiotic stress responses; for example, the foxtail millet bZIP transcription factor *SibZIP67* has been shown to enhance drought tolerance in *Arabidopsis thaliana* [20]. The *SiMYB16* gene has been shown to enhance plant salt tolerance through the modulation of lignin and suberin biosynthesis [21]. *SiSnRK2.6* has been shown to play a significant role in plant responses to low-potassium stress [22]. In contrast, the influence of biotic stresses on foxtail millet remains significantly less understood.

To elucidate the role of DAMPs in regulating foxtail millet root elongation and development, we treated foxtail millet with Pep1 and performed RNA sequencing for comprehensive transcriptome analysis. This approach provided valuable insights into the underlying molecular mechanisms through which this signaling peptide may regulate developmental and defense responses in a stress-tolerant cereal crop.

## 2. Results

### 2.1. Elicitor-Induced Immune Responses and Root Growth Inhibition

**Figure 1:**
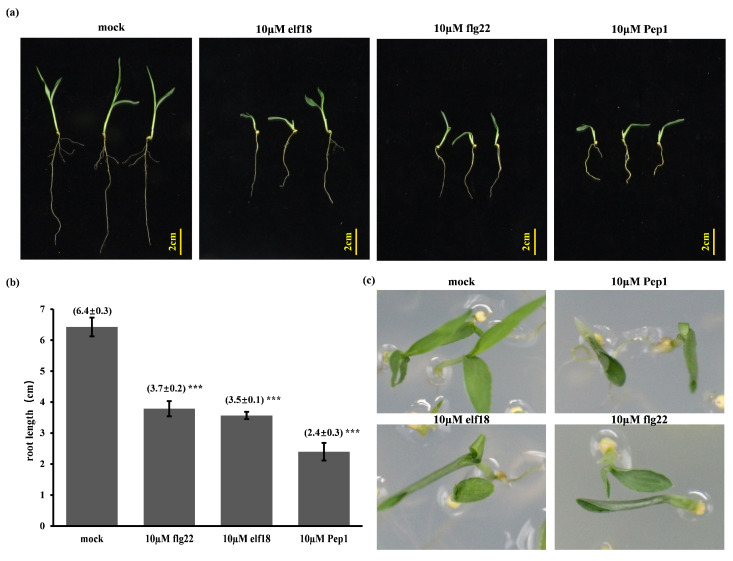
Elicitors flagellin epitope flg22, elongation factor Tu epitope elf18, and plant elicitor peptide (Pep), significantly inhibit primary root growth in Foxtail Millet. (**a**) Eight-day-old Foxtail Millet seedlings were continuously cultured under vertical orientation on Murashige and Skoog salts (MS) medium supplemented with 10 μM of the respective elicitors (flg22, elf18, or Pep1) or mock control. (**b**) Root length quantifications (mean ± SEM, *n* > 20; *** *p* < 0.001, ANOVA vs. mock) are presented. (**c**) Phenotypes of 8-day-old horizontally cultured foxtail millet seedlings with elicitors or mock control.

Elicitor treatments are recognized to activate plant PTI responses. Prior investigations in *Arabidopsis thaliana* and *Oryza sativa* [17,23,24] have indicated that the peptide elicitor Pep1 typically elicits a more substantial inhibitory effect on root growth compared to flg22 and elf18 under comparable exposure durations. To ascertain whether a similar differential sensitivity profile is evident in the C4 model species, foxtail millet (*Setaria italica*), we tested the impact of Pep1, in contrast to flg22 and elf18, on root length. At a concentration of 2 µM, none of the three tested short peptides demonstrated a statistically significant inhibitory effect on either root or shoot growth compared to the control (Figure S1). In light of the established resilience of foxtail millet to environmental stresses, we proceeded to examine higher concentrations, specifically 10 µM, to further explore potential inhibitory effects. Treatment with 10 µM elicitors significantly reduced primary root length in 8-day-old seedlings. Notably, Pep1 application resulted in a 90% reduction in root length (Figure 1a,b). To determine the temporal effects of elicitor exposure, 6-day-old seedlings were transferred to elicitor media, revealing that continuous exposure resulted in root growth inhibition similar to later transfers, and near-complete growth arrest with Pep1. Furthermore, 10 μM Pep1 treatment of 8-day-old horizontally grown seedlings significantly reduced both root and shoot length (Figure 1c). Collectively, these data demonstrate that Pep1 induces a markedly greater root growth inhibition than flg22 or elf18 in foxtail millet, indicating a distinct mechanism.

### 2.2. Synthetic Pep1 Inhibits Foxtail Millet Primary Root Growth

**Figure 2:**
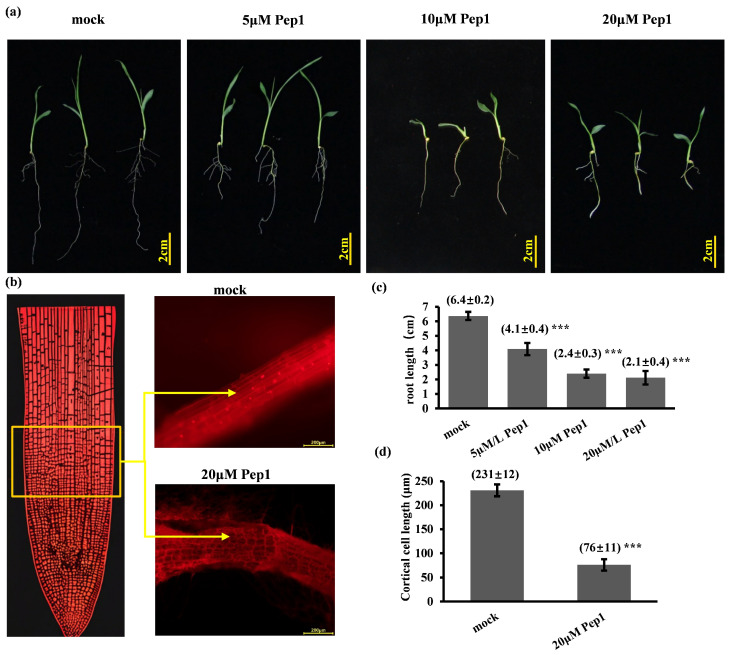
Pep1 inhibits foxtail millet primary root elongation. (**a**) Eight-day-old foxtail millet seedlings were continuously cultured under vertical orientation on MS medium supplemented with 5 μM, 10 μM, and 20 μM Pep1, with a mock control. (**b**) Longitudinal sections of root elongation zones: mock- and 20 μM Pep1-treated 8-day-old seedlings. Yellow box: sampling region; yellow arrows: elongated cortical cell. Scale bars = 200 μm. (**c**) Primary root length measurements of 8-day-old foxtail millet seedlings grown on media supplemented with 5 μM, 10 μM, and 20 μM Pep1, with a mock control. Scale bar = 2 cm. Root lengths: mean ± SEM, *n* > 20 (*** *p* < 0.001, ANOVA vs. mock). (**d**) Cortical cell length in root elongation zone: mean ± SD, *n* ≥ 15 (*** *p* < 0.001, *t*-test).

To evaluate the dose-dependent effects of Pep1 on foxtail millet growth, hydroponically grown seedlings were treated with a range of synthetic Pep1 concentrations (5–20 µM) for 8 days. Root growth inhibition increased in a dose-dependent manner within this range (Figure 2a), saturating at 20 μM. To investigate the cellular basis of Pep1-mediated root growth inhibition in foxtail millet, we performed propidium iodide (PI) staining of longitudinal root tip sections. Pep1 treatment resulted in significantly shortened cells in the elongation zone compared to mock-treated roots (Figure 2b). These results demonstrate that exogenous Pep1 suppresses foxtail millet root growth by inducing a curtailment of cortical cell elongation in the elongation zone.

### 2.3. Transcriptome Analysis of Foxtail Millet in Response to Pep1 Treatment

#### 2.3.1. Transcriptome Sequencing Data Quality Assessment

To investigate the molecular mechanisms of Pep1-induced root growth inhibition in foxtail millet, we performed RNA-seq on control and Pep1-treated samples. Raw read counts ranged from 36.9 to 44.1 million reads (Mb) per sample (Table S1). Following quality filtering, clean reads ranged from 35.8 to 43.4 million reads, with >90.79% alignment to the reference genome. The unique mapping rates were >90.43% (Pep1-treated) and >87.95% (control), and the Q20/Q30 values were >96% for all samples (Table S1). These metrics demonstrate high RNA-seq data quality, ensuring reliable downstream analysis (Table S1).

#### 2.3.2. Pep1-Mediated Different Gene Expression and GO Enrichment in Foxtail Millet Roots

**Figure 3:**
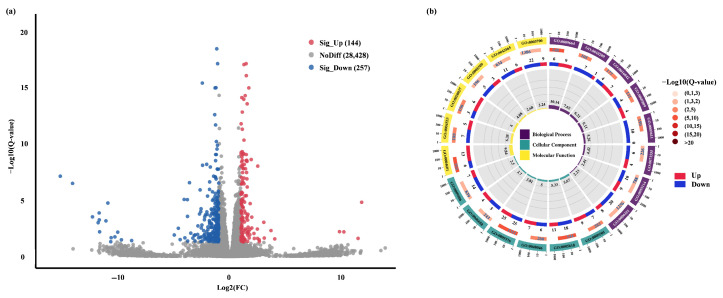
Gene Ontology (GO) enrichment analysis of differentially expressed genes (DEGs). (**a**) Volcano plot of DEGs between control and 10 μM Pep1-treated samples. (**b**) Top 20 enriched GO terms: nine molecular functions, six biological processes, and five cellular components.

To investigate Pep1-induced phenotypic changes at the transcriptional level, we compared differentially expressed genes (DEGs) between control and Pep1-treated samples. Pep1 treatment significantly altered gene expression, identifying 401 DEGs (Log_2_ FC ≥ 1 or ≤−1): 144 up-regulated and 257 down-regulated (Figure 3a, Table S2). Gene Ontology (GO) enrichment analysis, using the GO database (http://www.geneontology.org/, accessed on 25 May 2025), was performed to annotate the potential functions of the identified DEGs. GO analysis revealed the distribution of DEGs across three functional categories: 25 biological processes, 15 cellular components, and 10 molecular functions (Figure S2). Enrichment analysis (*p* ≤ 0.05) of genes associated with each GO term revealed 28 significantly enriched GO terms among the DEGs. Among the top 20 enriched GO terms, six were molecular functions, eight were biological processes, and six were cellular components (Figure 3b, Table S3). The top 20 GO terms (combined categories) showed significant enrichment for genes related to peroxidase activity, extracellular region, and cell wall, indicating their potential roles in the Pep1 stress response. In molecular functions (9 GO terms; Figure 3b, Table S3), DEGs were enriched in peroxidase activity, diacylglycerol O-acyltransferase activity, and heme binding. In biological processes (9 GO terms; Figure 3b, Table S3), DEGs were enriched in plant-type cell wall organization, cold acclimation, and lipid catabolic processes. To further explore the transcriptional landscape, we also performed a differential gene expression analysis using a wider threshold (Log_2_FC ≥ 0.6 or ≤−0.6), which resulted in an increased number of DEGs (1077 genes: 401 up-regulated, 676 down-regulated) (Figure S3). Notably, despite the increased number of DEGs identified with a relaxed threshold, the overall enriched GO terms and their ranking remained largely consistent with those identified using the stricter threshold.

#### 2.3.3. Transcriptional Regulation of Pep1 Response by Transcription Factors in Foxtail Millets

**Figure 4:**
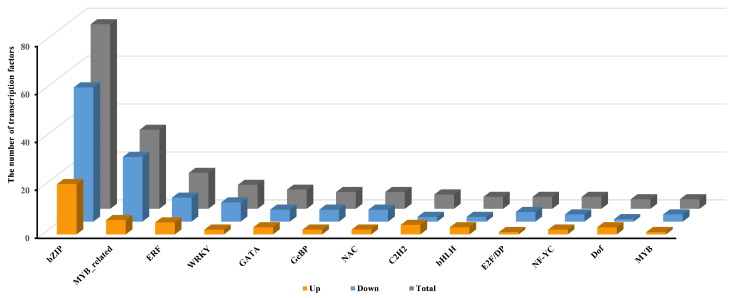
Distribution graphs of DEGs which belong to transcription factor gene families after following the 10 μM Pep1 treatment.

Considering the critical role of transcription factors (TFs) in plant stress responses, we analyzed TFs among the differentially expressed genes (DEGs) in Pep1-treated foxtail millet. Transcriptome analysis identified 205 differentially expressed TFs: 62 up-regulated and 143 down-regulated (Figure 4, Table S4). T The 205 differentially expressed TFs (27 gene families) were dominated by bZIP (77: 21 up, 56 down), MYB-related (33: 6 up, 27 down), ERF (15: 5 up, 10 down), WRKY (ten: two up, eight down), GATA (eight: three up, five down), NAC (seven: two up, five down), and other families (56: 23 up, 32 down) (Figure 4, Table S4). The bZIP family exhibited the greatest number of differentially expressed TFs, followed in descending order by MYB-related, ERF, and WRKY families (Figure 4, Table S4).

#### 2.3.4. Kyoto Encyclopedia of Genes and Genomes (KEGG) Pathway Analysis of Pep1 Stress Response

**Figure 5:**
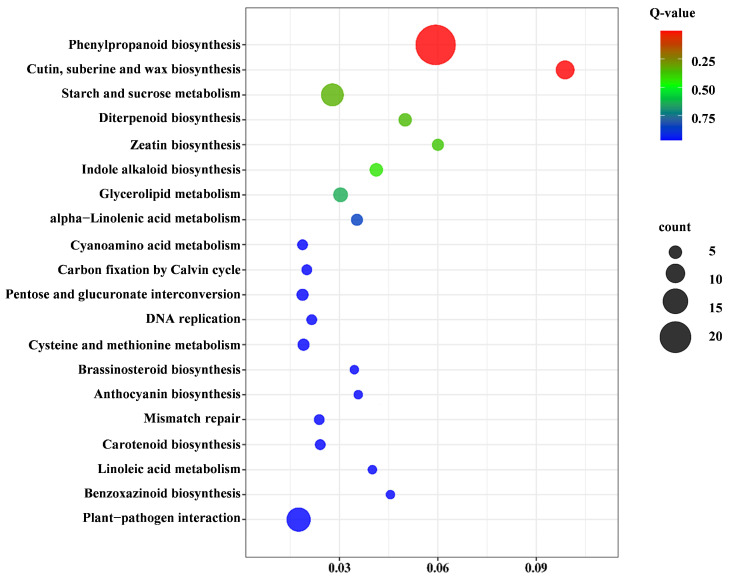
Kyoto Encyclopedia of Genes and Genomes (KEGG) analysis of the DEGs. The top 20 main pathways underlying 10 μM Pep1 stress were displayed.

To identify metabolic pathways affected by Pep1 stress, we performed KEGG pathway enrichment analysis of the differentially expressed genes (DEGs). This analysis revealed 292 DEGs significantly enriched in 87 pathways, with the top 20 shown in Figure 5. Six key KEGG pathways were significantly enriched for DEGs: phenylpropanoid biosynthesis (33 genes), starch and sucrose metabolism (26 genes), plant–pathogen interaction (16 genes), plant hormone signal transduction (13 genes), cutin, suberine, and wax biosynthesis (11 genes), and MAPK signaling pathway–plant (10 genes) (Table S5). These results indicate a crucial role for phenylpropanoid biosynthesis genes in the Pep1 stress response of foxtail millet.

### 2.4. qRT-PCR Validation and Functional Analysis of Pep1-Responsive DEGs

#### 2.4.1. qRT-PCR Validation

**Figure 6:**
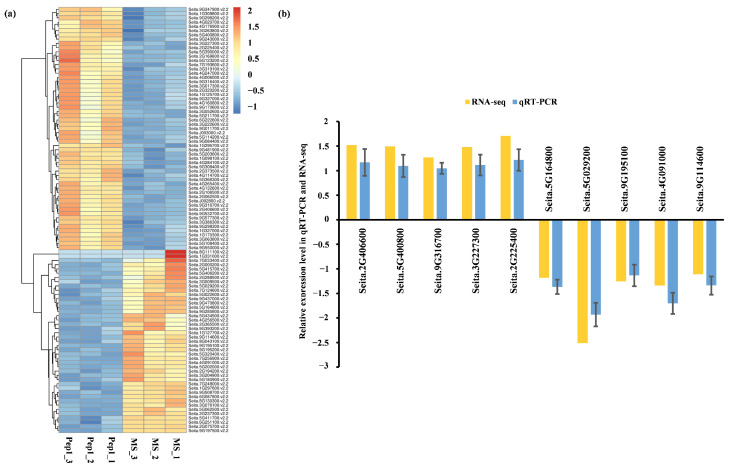
Heatmap illustrating the expression patterns of the top 100 DEGs in response to 10 μM Pep1 and mock treatment, along with qRT-PCR validation of selected DEGs. (**a**) Heatmap showing the expression pattern of the top 100 DEGs in 10 μM Pep1 and mock treatment. The color bar at the top right represents the relative expression value, with −1, 0, and 2 denoting low, medium, and high expression, respectively. (**b**) qRT-PCR validation of 10 randomly selected DEGs, *SiActin7* was used as normalizer.

To identify genes exhibiting significant expression changes in response to Pep1 treatment, the 100 genes with the greatest differential expression between Pep1-treated and mock-treated RNA-seq libraries were selected (Figure 6a). Subsequently, quantitative real-time PCR (qRT-PCR) analysis was conducted on 10 randomly selected differentially expressed genes (DEGs) (five up-regulated and five down-regulated) to confirm the reliability of the transcriptome data (Table S6). The qRT-PCR expression profiles of 10 DEGs matched the RNA-seq data (Figure 6), validating the transcriptome results.

#### 2.4.2. Pep1 Activates ROS-Mediated Immune Response and Pathogenesis-Related Protein 1 (PR1) Expression in Foxtail Millet Seedlings

**Figure 7:**
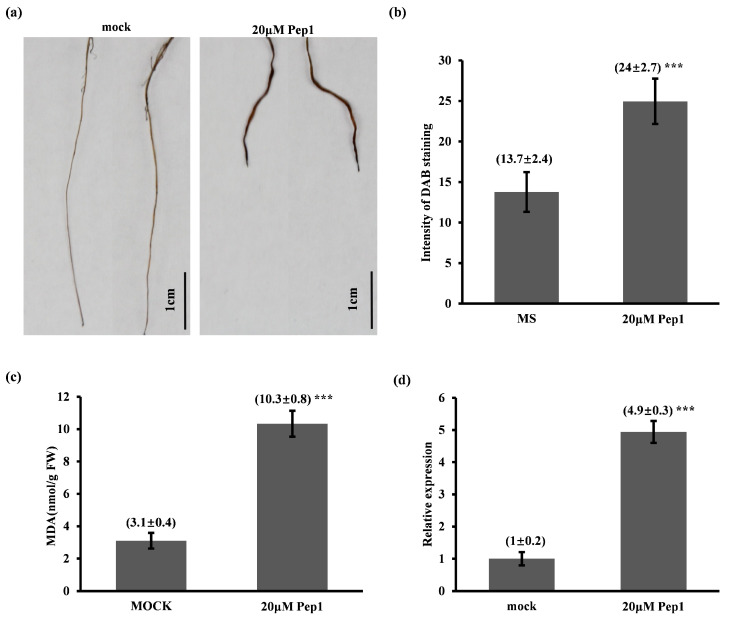
Effects of Pep1 treatment on root H_2_O_2_ homeostasis, oxidative stress, and pathogenesis-related protein 1 (*PR1*) transcript levels. (**a**) DAB staining of 8-day-old primary roots treated with mock solution or 20 μM Pep1. Scale bar = 1 cm. (**b**) Intensity of DAB staining the seedlings in panel (**a**). The histogram tool of ImageJ (version 1.54g, National Institutes of Health, USA) was used to record grayscale values of all pixels within the brown areas of eight-bit images. Data are shown as mean SD (*n* = 3). (**c**) Malondialdehyde (MDA) in foxtail millet roots. (**d**) *PR1* mRNA expression. Foxtail millet seedlings were treated with 20 μM Pep1 or water. After 12 h, *PR1* mRNA levels were measured by RT-qPCR analysis relative to mock plants. *SiActin7* was used for normalizer. Data are means ± SE of three biological repeats. (**b**–**d**) Data are presented as mean ± SD (*n* = 3, *** *p* < 0.001, ANOVA, LSD).

Transcriptome analysis identified ‘peroxidase activity’ as the most significantly enriched Gene Ontology (GO) category in response to Pep1 treatment, suggesting a potential role for reactive oxygen species (ROS) signaling. Given that hydrogen peroxide (H_2_O_2_), a key ROS, plays a critical role in modulating plant redox homeostasis and regulating root growth and development [25], we investigated whether Pep1 induces H_2_O_2_ accumulation. To this end, 8-day-old foxtail millet seedlings were subjected to 20 μM Pep1 treatment, followed by 3,3′-diaminobenzidine (DAB) staining to visualize H_2_O_2_ accumulation. As anticipated, Pep1 treatment resulted in a significant increase in H_2_O_2_ accumulation specifically within the root tissues (Figure 7a,b). To further investigate the potential for oxidative stress induced by Pep1, we quantified Malondialdehyde (MDA) levels, a well-established biomarker for lipid peroxidation. Consistent with the observed H_2_O_2_ accumulation, Pep1-treated seedlings exhibited a significant increase in MDA levels (Figure 7c). These findings provide compelling evidence that Pep1 triggers H_2_O_2_ accumulation in foxtail millet roots, potentially contributing to the observed root growth inhibition and immunity response.

Previous studies have shown that the expression levels of the pathogenesis-related protein 1 (*PR1*) gene in *Arabidopsis thaliana* can be significantly induced by flg22, elf18, and pep1. In light of this, we identified the *Arabidopsis thaliana PR1* gene homolog, *Seita.5G175100*, in foxtail millet. Sequence analysis revealed that this gene exhibits high conservation with *Arabidopsis thaliana PR1*, and it is also annotated as *PR1* in the foxtail millet genome database. Furthermore, reciprocal BLAST analysis using Phytozome’s online tool (https://phytozome-next.jgi.doe.gov/, accessed on 10 January 2025) confirmed that the best match for the *Seita.5G175100* gene in *Arabidopsis thaliana* is *PR1*, which is widely recognized as a classic marker gene for disease resistance in *Arabidopsis thaliana*. To further investigate the expression regulation of the *PR1* gene in foxtail millet, we examined changes in *PR1* transcript levels in foxtail millet seedlings treated with 20 μmol/L pep1. The results showed that *PR1* gene expression was significantly up-regulated compared to the control group (Figure 7d). These results suggest that Pep1 positively regulates PTI responses in foxtail millet.

## 3. Discussion

Elicitors flg22, elf18, and Pep1, recognized by FLS2, EFR, and PEPR1/2 receptors, respectively, trigger immune responses against pathogens [26,27,28]. While signaling peptides are known to regulate root growth and development [29], and several root-regulating peptides have been identified in *Arabidopsis* and rice [17,30], their roles in foxtail millet remain largely unknown. We observed that Pep1 strongly inhibited foxtail millet root growth compared to flg22 and elf18, consistent with previous findings in *Arabidopsis* and rice [16,17]. To elucidate the Pep1 signaling pathways involved in root growth and disease resistance, we performed transcriptome analysis.

GO enrichment analysis highlighted peroxidase activity as a major biological function associated with Pep1 treatment in foxtail millet. Consistent with this, DAB staining revealed H_2_O_2_ accumulation in Pep1-treated roots (Figure 6a,b). These results suggest that Pep1 acts as a damage-associated molecular pattern (DAMP), activating ROS signaling, consistent with its known role in eliciting plant immune responses [31]. Peroxidases, key enzymes in ROS regulation, indicate Pep1 triggers a strong defense response through redox modulation. ROS, including H_2_O_2_, function as both signaling molecules and antimicrobial agents [32]. Our data suggest that Pep1-induced ROS accumulation contributes to defense gene expression and root growth inhibition, supported by observed morphological and transcriptional changes. Further studies on specific peroxidases and their regulation will elucidate Pep1-mediated defense signaling in foxtail millet.

KEGG pathway enrichment analysis revealed that Pep1 stress significantly altered metabolic pathways in foxtail millet, enriching phenylpropanoid biosynthesis, plant–pathogen interaction, and plant hormone signal transduction. This indicates that Pep1 induces a complex defense response involving secondary metabolite production, immune signaling, and hormonal modulation for stress adaptation. Phenylpropanoid biosynthesis, crucial for cell wall reinforcement and ROS scavenging, likely strengthens physical barriers and mitigates oxidative damage, consistent with DAMP responses [33,34]. Activation of the plant–pathogen interaction pathway suggests Pep1 triggers pathogen-triggered immunity (PTI)-like signaling, supporting DAMPs’ ability to elicit PTI-like responses [35]. Plant hormone signaling, involving JA and ET, may coordinate growth adjustments and defense activation [36]. Additionally, enrichment of starch/sucrose metabolism and MAPK signaling indicates that energy mobilization and signal transduction are essential for Pep1 response [37]. In summary, KEGG analysis highlights Pep1-induced metabolic shifts in foxtail millet, emphasizing the complexity of its defense response. Future studies should validate specific gene functions, regulatory networks, and pathway crosstalk to fully understand Pep1’s impact.

Transcriptome analysis revealed significant enrichment of bZIP TFs among differentially expressed genes (DEGs). Given bZIP TFs’ known roles in stress responses, including pathogen defense and oxidative stress [38], their enrichment suggests that they regulate Pep1-induced gene expression. bZIP TFs modulate genes involved in ROS detoxification, hormone signaling, and defense [39], implying that they are key regulators of the Pep1 pathway, mediating signal transduction through target gene modulation. Future studies will identify specific bZIP target genes and their interactions with other signaling components to elucidate Pep1-mediated gene expression.

Our findings reveal that exogenous Pep1 application in foxtail millet seedlings triggers a rapid accumulation of hydrogen peroxide (H_2_O_2_) in root tissues, a hallmark of early stress responses. This oxidative burst is further corroborated by elevated levels of lipid peroxidation, as indicated by increased malondialdehyde (MDA) content, suggesting the induction of oxidative stress. Consistent with these biochemical changes, our transcriptome analysis highlighted a significant enrichment of genes encoding peroxidases, enzymes critical in ROS metabolism. Given the well-established role of H_2_O_2_ as a key reactive oxygen species (ROS) involved in regulating plant redox homeostasis and its documented influence on fundamental root developmental processes such as cell wall remodeling and cell elongation, we propose that Pep1-induced H_2_O_2_ accumulation represents a plausible mechanism contributing to the observed inhibition of root growth in foxtail millet.

Consistent with the effects on oxidative stress and root development, Pep1 treatment also led to a significant up-regulation of the pathogenesis-related protein 1 (*PR1*) gene in foxtail millet leaves. The *PR1* gene is a widely recognized molecular marker for systemic acquired resistance (SAR) in *Arabidopsis thaliana*, and its transcriptional induction is generally associated with enhanced resistance to a broad spectrum of pathogens. Notably, our results align with previous studies demonstrating that various plant-associated molecular patterns (PAMPs), including flg22, elf18, and, importantly, Pep1 itself, can potently induce *PR1* expression in *Arabidopsis thaliana*. This conservation of *PR1* induction suggests a potentially conserved signaling pathway mediating Pep1-triggered immune responses across plant species.

Collectively, our data indicate that Pep1 elicits a dual response in foxtail millet seedlings: the induction of an oxidative burst in roots, potentially contributing to growth inhibition, and the activation of *PR1* gene expression in leaves, indicative of systemic immune activation. These findings suggest that Pep1, acting as a damage-associated molecular pattern (DAMP), may trigger defense responses in foxtail millet through the modulation of ROS signaling pathways while simultaneously influencing root architecture, possibly also via ROS-mediated mechanisms. The observed root growth inhibition, characterized by reduced meristem size and cortical cell elongation, is consistent with DAMP effects reported in other plant species [40], and the concurrent H_2_O_2_ accumulation in roots provides a potential mechanistic link, given the known role of ROS in regulating cell division and elongation [41].

Future research should focus on dissecting the intricate signaling network downstream of Pep1 perception in foxtail millet. Investigating the temporal dynamics of key defense-related genes, cell cycle regulators, and cell elongation-specific genes will provide a more comprehensive understanding of the molecular mechanisms underlying Pep1-mediated root development modulation and immune activation. Additionally, identifying the specific receptors and downstream signaling components involved in these distinct yet potentially interconnected responses will be crucial for a holistic understanding of Pep1 function in this stress-tolerant cereal crop. Ultimately, these investigations will contribute valuable insights into plant immunity and development, potentially informing strategies for enhancing crop resilience.

## 4. Materials and Methods

### 4.1. Plant Materials and Growth Conditions

Foxtail millet seeds (*Setaria italica* cv. Jingu21), obtained from Professor Xingchun Wang (College of Agriculture, Shanxi Agricultural University, Taigu, China)were surface-sterilized with 2.5% plant preservative mixture (Caisson Laboratories, Smithfield, UT, USA) and stratified at 4 °C for 3 days in the dark. Subsequently, seeds were transferred to 100 mm × 100 mm round tissue culture flasks containing half-strength Murashige and Skoog salts (MS; Gibco, Waltham, MA, USA), supplemented with 1.5% (*w*/*v*) sucrose (Sigma, St. Louis, MO, USA) and 0.25% (*w*/*v*) agar (Becton Dickinson, Franklin Lakes, NJ, USA), pH 6.0 (adjusted with KOH). The plants were grown in a controlled environment chamber at 28 °C/24 °C (day/night) with a 16 h light/8 h dark photoperiod. The treatment solutions of flg22, elf18, and Pep1 [32,42,43] were synthesized by QYAOBIO (Chinapeptides Co., Ltd., Shanghai, China).

### 4.2. RNA-Seq Analysis

Foxtail millet seedling roots, subjected to mock (H_2_O) and 20 μM Pep1 treatments, were excised and immediately frozen in liquid nitrogen. The samples were stored at −80 °C until RNA extraction. For each treatment, three biological replicates, each consisting of ten independent seedlings, were prepared. RNA-seq was performed by LC-BIO TECHNOLOGIES (Hangzhou, China) using the Illumina platform. Reads were mapped to the *Setaria italica* reference genome (https://phytozome-next.jgi.doe.gov/, accessed on 15 August 2024) using HISAT2 [44], and gene annotation information was generated using featureCounts [45]. Transcript expression levels were normalized as fragments per kilobase of transcript per million mapped reads (FPKM), calculated as follows: FPKM = C × 10^9^/(N × L), where C is the number of fragments mapped to a transcript, N is the total number of mapped fragments, and L is the transcript length [46]. Differential gene expression analysis was conducted using the R package edgeR [47], with a false discovery rate (FDR) correction to account for multiple comparisons. Genes with an FDR ≤ 0.05 and an absolute Log_2_ fold change (Log_2_FC) ≥ 1 were considered statistically significant [48].

### 4.3. GO and KEGG Analysis

Differentially expressed genes (DEGs) were subjected to Gene Ontology (GO) and Kyoto Encyclopedia of Genes and Genomes (KEGG) 1 enrichment analyses. GO enrichment was performed using Goatools, with Fisher’s exact test, to identify statistically significant GO terms (Benjamini–Hochberg-corrected Q-value (*P_adj_*) ≤ 0.05). KEGG pathway enrichment was conducted using the KEGG database (https://www.kegg.jp/kegg/, accessed on 10 September 2024) to identify enriched metabolic pathways, employing the same statistical methodology as the GO enrichment analysis.

### 4.4. Validation of the DEGs by Quantitative Real-Time PCR

Quantitative real-time PCR (qRT-PCR) was performed on 17 randomly selected differentially expressed genes (DEGs) to validate the RNA-seq data. An ABI 7500 Real-Time PCR System (Applied Biosystems, Waltham, Massachusetts, USA) was used for qRT-PCR. SiActin7 (*Seita.7G294000*) served as the internal control for normalization. Each experiment consisted of three biological replicates, with each biological replicate assayed in technical triplicate. Relative gene expression levels were calculated using the 2^−(ΔΔCT)^ method [49].

### 4.5. DAB Staining and Malondialdehyde (MDA) Content

H_2_O_2_ accumulation in 8-day-old foxtail millet root tissues, treated with 10 μM Pep1, was visualized using 3,3′-diaminobenzidine (DAB) staining. The seeds were germinated for two days on solid Murashige and Skoog (MS) medium, with or without Pep1. The roots were subsequently rinsed, immersed in 0.1% (*w*/*v*) DAB solution (pH 3.8), and subjected to 5 min vacuum infiltration (0.08 MPa). After 12 h of dark incubation at 25 °C, DAB staining intensity, reflecting H_2_O_2_ levels, was quantified using ImageJ. Grayscale pixel values within DAB-H_2_O_2_ reaction product areas were measured from eight-bit images, applying a uniform noise threshold across all replicates. Three biological replicates were performed. The MDA content in whole plants was examined using an MDA assay kit (G0109W, Grace Biotechnology, Suzhou, China) according to the manufacturer’s instructions.

## Data Availability

The original contributions presented in this study are included in this article; further inquiries can be directed to the corresponding author.

## Supplementary Materials

The following supporting information can be downloaded at: (https://drive.google.com/file/d/1PZIG1gc039TCGBqfcmKs2lCvegBype9H/view?usp=sharing, accessed on 17 May 2025).
