# Supplementary figures and images for: Transcriptome Analysis of DAMP-Induced Root Growth Regulation and Defense in Foxtail Millet
Source: Int J Mol Sci. 2025 May 28;26(11):5175. doi: 10.3390/ijms26115175 (PMC12154379; doi:10.3390/ijms26115175)

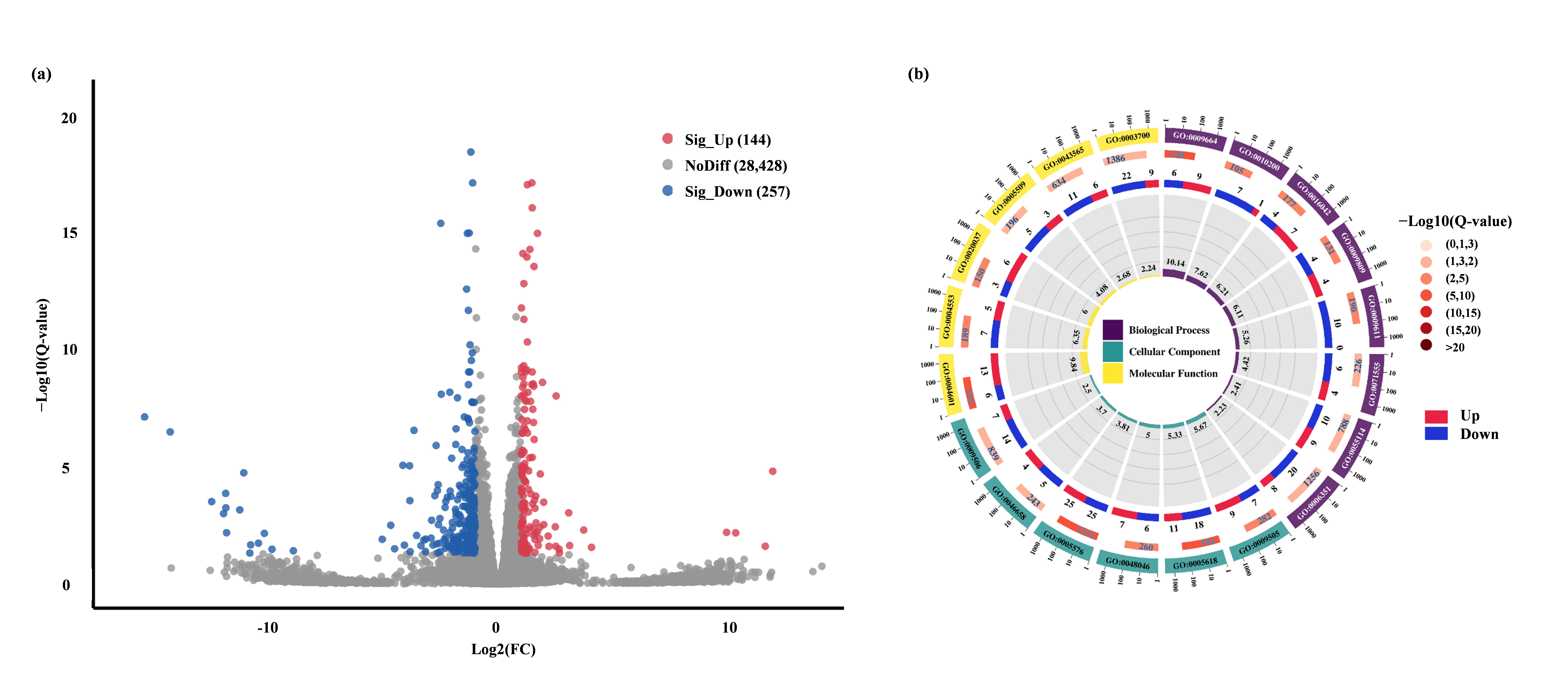

Supplement: Supplementary file 1 [file ijms-26-05175-s001.zip › FIG3,FIG6/FIG3.jpg]

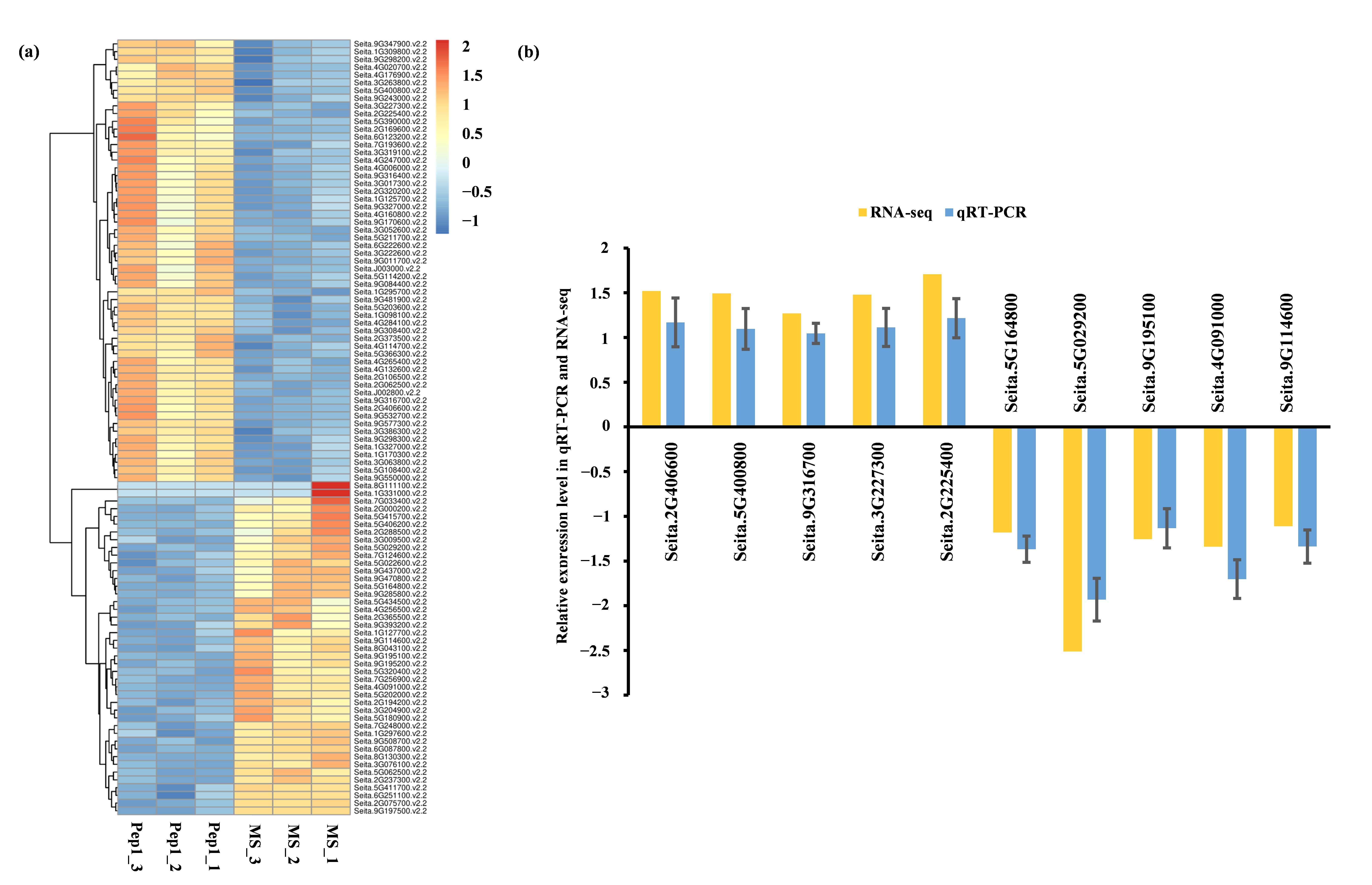

Supplement: Supplementary file 1 [file ijms-26-05175-s001.zip › FIG3,FIG6/FIG6.jpg]
